# 1305. Current Management of Osteoarticular Infections by Infectious Diseases Physicians: Results of an Emerging Infections Network (EIN) Survey

**DOI:** 10.1093/ofid/ofad500.1144

**Published:** 2023-11-27

**Authors:** Nicolas W Cortes-Penfield, Susan E Beekmann, Philip M Polgreen, Keenan L Ryan, Poorani Sekar

**Affiliations:** University of Nebraska Medical Center, Omaha, Nebraska; University of Iowa, IOWA CITY, Iowa; University of Iowa Carver College of Medicine, Iowa City, IA; University of New Mexico Hospitals, Albuquerque, NewMexico; University of Iowa Hospitals and Clinics, Iowa City, Iowa

## Abstract

**Background:**

Osteoarticular infections (OAI) have been commonly treated with prolonged intravenous (IV) antimicrobials. The Oral versus Intravenous Antibiotics for Bone and Joint Infection (OVIVA) trial demonstrated that oral (PO) antibiotic therapy was noninferior to IV antibiotics in the treatment of OAIs. We surveyed Infectious disease (ID) physicians to see how often they used PO antibiotics in the treatment of OAIs and the barriers to using PO antibiotics.

**Methods:**

An EIN survey was sent to 1475 ID physicians. The survey had 9 questions pertaining to antibiotic prescribing patterns in the treatment of OAIs. The questions were mostly multiple choice, Yes/No questions with an area provided for free text. Definitive oral antibiotic therapy was defined as switching to PO antibiotics within 2 weeks of starting antibiotics.

**Results:**

There were 413 respondents who treated OAIs and responded to the survey. A plurality of respondents 150/413 (36%) switched from IV to PO antibiotics after 4 weeks of IV therapy. 129/413 (31%) switched to oral antibiotics as definitive treatment (Fig 1). PO antibiotics were used as definitive therapy most often for diabetic foot osteomyelitis and native joint septic arthritis (Fig 2). Trimethoprim-sulfamethoxazole was most often used as definitive therapy for *Staphylococcus aureus* infections followed by doxycycline/minocycline. Amoxicillin/ cefadroxil/ cephalexin was used for treatment of streptococcal infections and fluoroquinolones were used as definitive treatment for infections caused by Gram-negative organisms. The most common reasons for not transitioning to PO antibiotics included non-susceptible pathogen, comorbidities preventing therapeutic drug levels and concern about adherence

**Figure:**
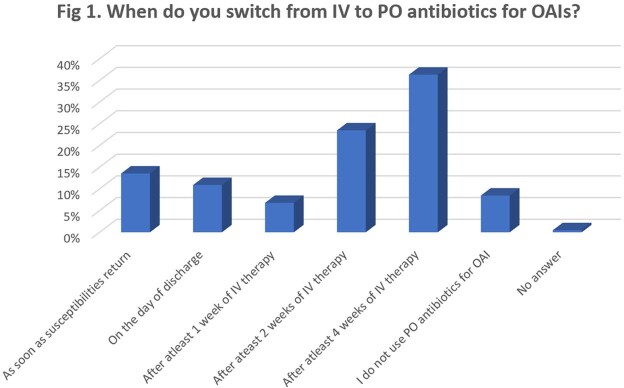

**Figure:**
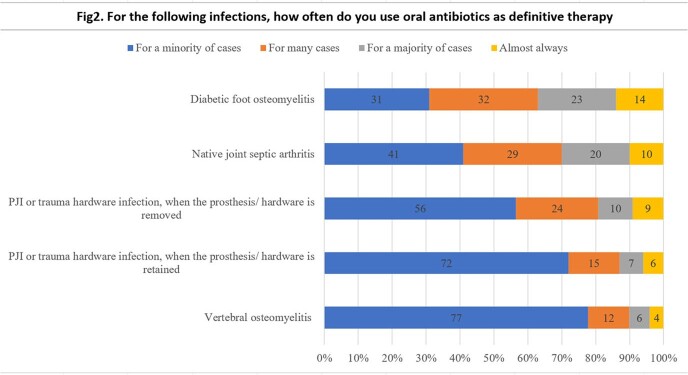

For the following infections, how often do you use oral antibiotics as definitive therapy?

**Conclusion:**

A sizeable portion of ID physicians utilize PO antibiotics for the management of OAIs, although significant practice variation exists regarding the timing of IV to PO switch.

**Disclosures:**

**Philip M. Polgreen, MD**, Eli Lilly: Case adjudication for a clinical trial **Keenan L. Ryan, PharmD, PhC**, PharmCon: Honoraria

